# A sensitive bioassay to measure NOD1/2 ligands in human serum reveals differential postprandial NOD2 activation

**DOI:** 10.3389/fnut.2025.1596951

**Published:** 2025-06-25

**Authors:** Lucy Biber, Nadine Schart, Anja Bosy-Westphal, Thomas A. Kufer

**Affiliations:** ^1^Department of Immunology, Institute of Nutritional Medicine, University of Hohenheim, Stuttgart, Germany; ^2^Institute of Human Nutrition and Food Science, Kiel University, Kiel, Germany

**Keywords:** reporter gene assay, NLR, peptidoglycan, food intake, immune response, innate immunity, SEAP

## Abstract

Food intake is associated with the occurrence of components and metabolites from the gut microbiota in the bloodstream. Using a widely utilised cell-based assay to measure bacterial peptidoglycan via pattern-recognition receptor activation, we found that the performance of this assay is significantly influenced by the presence of other serum components. To address this challenge, an alternative luciferase-based reporter assay protocol was established to accurately measure NOD1 and NOD2 activation by serum samples with high sensitivity. Utilising postprandial human serum samples, we tested this assay and showed that the concentration of NOD2-activating ligands differed in the postprandial phase. Together, we provide a protocol to measure NOD1/2 activation by human serum samples and highlight a role for NOD2 in the postprandial response.

## Introduction

The intricate interplay between nutrition and the gut microbiota has emerged as a critical area of research, particularly in elucidating how dietary components influence microbial composition and function, as well as their systemic effects on human health (1). Within the immune system, nucleotide binding oligomerization domain containing 1 (NOD1) and nucleotide binding oligomerization domain containing 2 (NOD2) are pivotal pattern recognition receptors (PRRs) that detect bacterial components and initiate innate immune responses. NOD1 is primarily activated by γ-D-glutamyl-meso-diaminopimelic acid (iE-DAP), a peptidoglycan fragment found predominately in Gram-negative bacteria, while NOD2 recognizes muramyl dipeptide (MDP), a conserved motif found in the peptidoglycan structure of both Gram-positive and Gram-negative bacteria (2). Upon ligand recognition, these receptors activate downstream signaling pathways, including nuclear factor kappa B (NF-κB) and mitogen-activated protein kinases (MAPKs), leading to the production of pro-inflammatory cytokines, chemokines, and antimicrobial peptides. These responses are essential for host defense against microbial infections and play a crucial role in maintaining gut homeostasis (3).

Dysregulation of NOD1 and NOD2 signaling has been implicated in inflammatory bowel diseases (IBD), metabolic syndrome, and other immune-related disorders (4). Notably, diet has a profound impact on immune function, both long lasting and at early time points through a process termed postprandial inflammation. Following food intake, transient absorption of dietary components such as lipids and carbohydrates triggers low-grade inflammation characterized by elevated serum levels of interleukin-6 (IL-6) (5). This postprandial response has been associated with an increased risk of chronic diseases such as cardiovascular disease and insulin resistance (6). The mechanisms underlying postprandial inflammation are discussed to be multifactorial and seem to involve interactions between dietary components and the gut microbiota. Translocation of microbial-associated molecular patterns (MAMPs), including lipopolysaccharides (LPS) and peptidoglycan fragments, into systemic circulation is one possible cause of this inflammatory response. Some members of the NLR-family of proteins, including NOD1 and NOD2 have been shown to contribute to the postprandial inflammatory milieu, and can contribute to exacerbate inflammation and to metabolic complications like insulin resistance (7, 8).

To investigate physiological activation of NOD1 and NOD2, cell-based reporter assays have become indispensable tools. These assays typically utilize cell lines transfected with plasmids encoding NOD1 or NOD2 to measure receptor activation upon exposure to specific ligands. Two widely used methods are the secreted embryonic alkaline phosphatase (SEAP) assay and the luciferase reporter assay. The SEAP assay quantifies alkaline phosphatase activity secreted into the medium under promoter control responsive to NOD signaling pathways like NF-κB (9). In the luciferase reporter assay, light emission catalyzed by luciferase under similar promoter control is used as readout, offering high sensitivity suitable for high-throughput applications (10).

In this study, we provide evidence that SEAP-based reporter assays are not well suited for measuring serum NOD1/2 ligands. To overcome these limitations, we established a protocol for an optimized luciferase-based reporter assay to measure NOD1 and NOD2 activation by bioactive bacterial peptidoglycan in human serum samples. Validation experiments confirmed good correlations between luminescence signals and concentrations of known ligands for these receptors. By examining fluctuations in NOD2 activation by serum in response to dietary intake our findings provide novel insights into how nutrition modulates immune function in humans.

## Methods

### Human serum samples

Human serum from eight participants of the meal skipping study (11), which has been stored at −80°C, was used. In brief, blood was drawn before (0 h) and 0.5 h, 1 h, 1.5 h, 2 h, and 4 h after lunch following a normal breakfast (from the dinner skipping day) or skipped breakfast (S1–S8). The study protocol was approved by the ethics committee of the Medical Council of Baden-Württemberg, Germany. The trial is registered at clinicaltrials.gov as NCT02635139. For establishment of the assays, human sera from three healthy individuals were used, which was approved by the ethics committee of the University of Hohenheim (09.06.2021). Studies were conducted in accordance with the Declaration of Helsinki and all participants provided written informed consent before participation.

### SEAP-based HEK-Blue NF-κB reporter assay

Per well, 5 × 10^4^ HEK-Blue™ hNOD1 or HEK-Blue^™^ hNOD2 cells (InvivoGen) were seeded in 90 μL DMEM (Gibco, Thermo Fisher Scientific) with 10% heat treated (20 min at 56°C) FBS Xtra (Capricorn) in an F-bottom 96-well plate (Greiner Bio-One). Cells were stimulated with 10 μL human serum, DMEM as unstimulated control or the indicated concentration of MDP (tlrl-mdp, InvivoGen) and TriDAP (tlrl-tdap, InvivoGen). After 16 h at 37°C with 5% CO2 in a humid atmosphere, 20 μL of the supernatant was added to 180 μL QUANTI-Blue^™^ solution (InvivoGen). Absorbance at 620 nm was measured photometrically (EnSpire, Perkin Elmer) after 7 h at 37°C and normalized to the absorbance of unstimulated cells to calculate relative NF-κB activity. Cells were maintained in the presence of 30 μg/mL blasticidin (Invivogen) and 100 μg/mL Zeocin (Invivogen).

To test for alkaline phosphatase activity in human serum samples, 20 μL of serum was incubated with 180 μL QUANTI-Blue solution and measured after 2 h at 37°C with the same settings as described above.

### NF-κB luciferase reporter assay

NF-κB luciferase reporter assays were performed as described in Zurek et al. (10). In brief 3 × 10^4^ HEK293T cells were seeded in 50 μL DMEM with 10% FBS and 20 μL DMEM per well in an F-bottom 96-well plate and incubated for 1 h at 37°C with 5% CO_2_ in a humid atmosphere. For transfection, plasmids in 20 μL OptiMEM (Gibco, Thermo Fisher Scientific) were incubated with 0.17 μL XtremeGene9 (Roche) for 20 min at room temperature. Per well, plasmid mixture consisted of: 13 ng luciferase under an NF-κB promoter, 8.6 ng constitutively expressed β-galactosidase and when indicated 0.25 ng NOD1 or 0.05 ng NOD2, ad 51 ng pcDNA.

Directly or, if not indicated elsewise, 10 h after transfection for serum stimulation experiments, cells were stimulated with indicated concentrations of MDP, TriDAP or human serum. DMEM served as unstimulated control. Readout was performed 16 h or 14 h after stimulation for serum experiments as described in Zurek et al. (10) and Kuri et al. (12). In brief, cells were lysed and luciferase activity as a readout for NF-κB activity (RLU) and β-galactosidase activity were measured. If not stated otherwise, NF-κB activity was normalized to β-galactosidase signal (nRLU). Relative NF-κB activity was calculated by the ratio of the normalized stimulated to the normalized unstimulated cell NF-κB activity.

### Statistics

Statistic analysis was performed with Python 3 (Python Software Foundation) and the help of scipy and scikit-posthocs packages. To analyze postprandial differences in NOD1/NOD2 ligands, the Kruskal–Wallis test was used, followed by Dunn’s test if significant. Data is presented as mean with standard error of mean (SEM). *p*-values below 0.05 were considered statistically significant.

## Results

### Functionality of SEAP-based cellular bioassay for measurements of human serum samples

Commercially available reporter cell lines for human NOD1 and NOD2 (HEK-Blue cell lines; InvivoGen) testing stably express the human PRR NOD1 or NOD2 and a SEAP reporter under control of the NF-κB promotor. Upon ligand recognition, NOD1 and NOD2 are activated and induce activation of the transcription factor NF-κB, resulting in the transcription and secretion of SEAP in the supernatant, which can subsequently be detected by the SEAP detection reagent QUANTI-Blue (InvivoGen).

**Figure 1:**
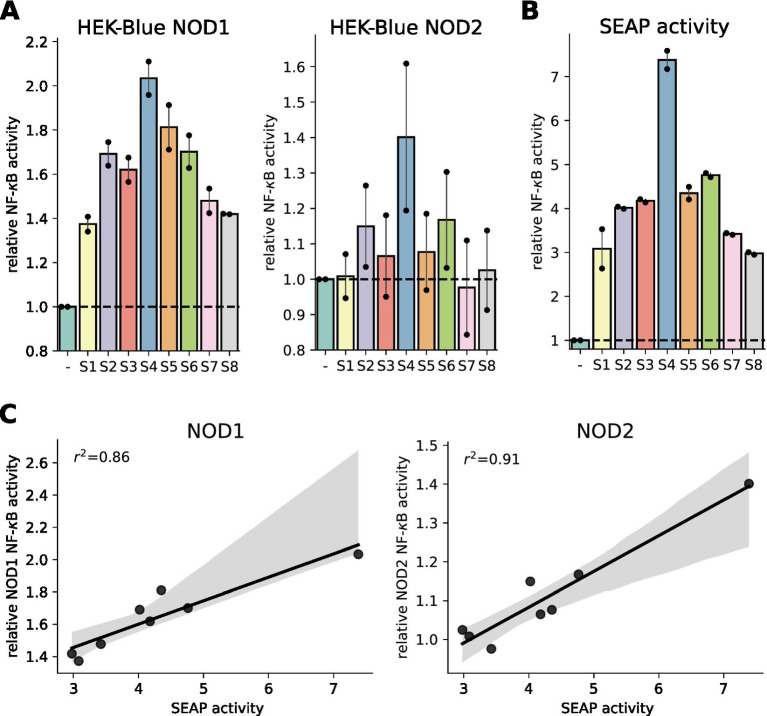
Characterization of SEAP-based HEK-blue assays to measure NOD1 and NOD2 activation in serum samples. **(A)** NF-κB activation potential of human serum (S1 to S8) 1.5 h after lunch on the breakfast-skipping day was measured with HEK-Blue NOD1 and HEK-Blue NOD2 cells. The signal was normalized to unstimulated cells. **(B)** Alkaline phosphatase activity was determined by incubating human serum with QUANTI-Blue detection solution and normalization to QUANTI-Blue signal alone. **(C)** Linear regression of NOD1/NOD2 HEK-Blue cell measured NF-κB activation potential and alkaline phosphatase activity of human serum. 95% confidence interval and squared Pearson correlation coefficient are shown. Two independent experiments were performed. Dots represent mean of technical triplicates for each experiment and bars represent mean and SEM of both runs together.

This assay is frequently used, most recently in a study showing that in mice, a high-fat diet led to an increase in NOD1 ligands in the serum (13). Here, we tested human serum of eight participants of the meal skipping study (11) using the human NOD1 and NOD2 HEK-Blue cell lines. For both PRRs, we observed consistent differences between the individuals (Figure 1A). As human serum contains alkaline phosphatase (AP) (14), we wondered if the human enzyme could catalyze the detection reagent as well. To this end, we incubated the serum with the detection reagent and observed a similar activation pattern (Figure 1B), which showed a high correlation (squared Pearson correlation coefficient of 0.86 for NOD1 and 0.91 for NOD2) with the measured NF-κB activation of the NOD1 and NOD2 cell lines (Figure 1C). This showed that the measured signal in the reporter assay is likely explained by different AP concentrations in the serum samples rather than by differences in NOD1/2 agonists. This shows, that a SEAP-based reporter system is not well suited to detect PRR activity induced by serum from humans.

### Development of a protocol for a robust luciferase-based reporter assay to measure NOD1 and NOD2 activation in human serum samples

**Figure 2:**
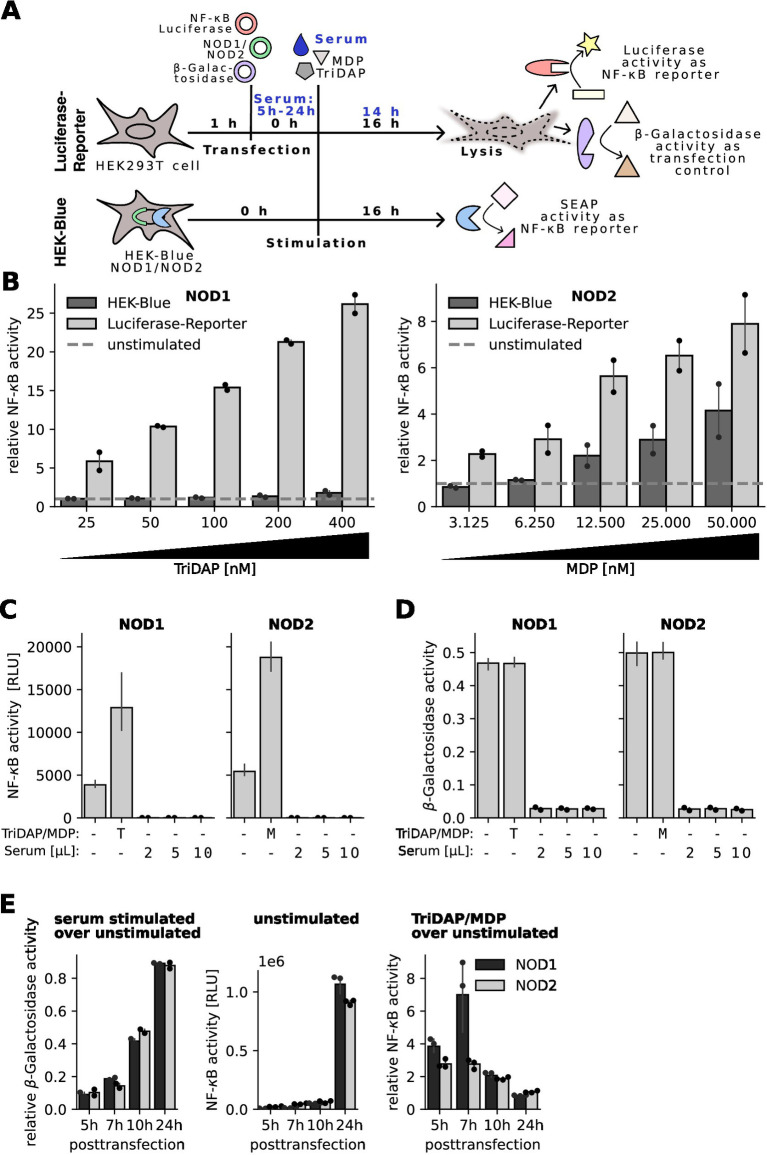
Characterization of an optimized protocol for an NF-κB luciferase reporter assay. **(A)** Schematic overview of the luciferase and HEK-Blue NF-κB reporter assay. **(B)** HEK-Blue NOD1/NOD2 cells or HEK293T cells transfected with the NOD1/NOD2 luciferase reporter system were stimulated with increasing concentrations of TriDAP or MDP, respectively. NF-κB signal was normalized to unstimulated cells. Dashed line represents unstimulatedcells. Graphs show mean and SEM of two independent experiments performed in technical triplicates. **(C,D)** NOD1 and NOD2 luciferase reporter assays were stimulated directly after transfection with different amounts of two different human sera, indicated as dots, and 20 nM TriDAP (T) or 10 nM MDP (M) as positive control. **(C)** Shows measured luciferase signal for NF-κB activation and **(D)** β-galactosidase activity as transfection control. **(E)** NOD1 and NOD2 luciferase reporter assay was performed with stimulation 5 h, 7 h, 10 h, and 24 h after transfection. (Left) β-galactosidase signal of serum stimulated cells was normalized to β-galactosidase signal of unstimulated cells of the same plasmid type and posttransfection time point. (Middle) Raw NF-κB activity of unstimulated cells is depicted. (Right) NOD1 and NOD2 transfected cells were stimulated with 40 nM TriDAP or 20 nM MDP, respectively. Ligand stimulated NF-κB activity was normalized to NF-κB activity of unstimulated cells. Bars represent mean and SEM of technical triplicates from one experiment.

To overcome these limitations, we switched to the widely used luciferase reporter-gene assay (10, 12). This system relies on transient transfection of human HEK293T cells with a luciferase reporter plasmid under the control of a strong NF-κB-inducible promotor, a constitutively active β-galactosidase expression plasmid and expression plasmids encoding the PRR of interest (Figure 2A upper panel). To compare this assay with the HEK-blue assay, TriDAP and MDP were titrated in the NOD1 and NOD2 transfected cells, respectively. As expected, both assays showed a dose-dependent response towards the respective ligand, however the luciferase reporter assay turned out to be more sensitive. The NOD1 luciferase reporter assay already detected 25 nM TriDAP, whereas the HEK-blue NOD1 cell line only showed a slight increase in NF-κB-activity with 400 nM TriDAP (Figure 2B).

Next, we added different amounts of human serum to the NOD1 and NOD2 transfected cells. Already using 4% of serum thereby led to complete block of basal luciferase activity (Figure 2C). In line, no β-galactosidase activity was detected in the serum treated samples Figure 2D. This suggested that the serum either interfered with the enzymatic detection or the transfection procedure. To clarify this, serum was added 5 h, 7 h, 10 h, and 24 h after transfection, respectively. The β-galactosidase activity of the serum stimulated cells increased with longer incubation periods between transfection and serum stimulation (Figure 2E left panel). On the other hand, we observed an increased auto-activity of both PRRs with increased transfection times, depicted by increased luciferase (NF-κB)-activity of the unstimulated cells (Figure 2E middle panel). The RLU values obtained with the 24 h transfection reached the detection limit, impairing interpretation of NF-κB activation by potential NOD1/2 agonists. The β-galactosidase activity, representing a proxy for transfection efficacy and cell viability, was very low up to 7 h posttransfection but increased after 10 h and 24 h of incubation. With the 10 h incubation time we could obtain a good compromise between robustness and sensitivity of the assay, showing for NOD2 a 1.4-fold higher sensitivity compared to 5 h (Figure 2E right panel). We thus decided to use a 10 h incubation period for further assays.

### Sensitivity of the assay and freeze-thaw effect

**Figure 3:**
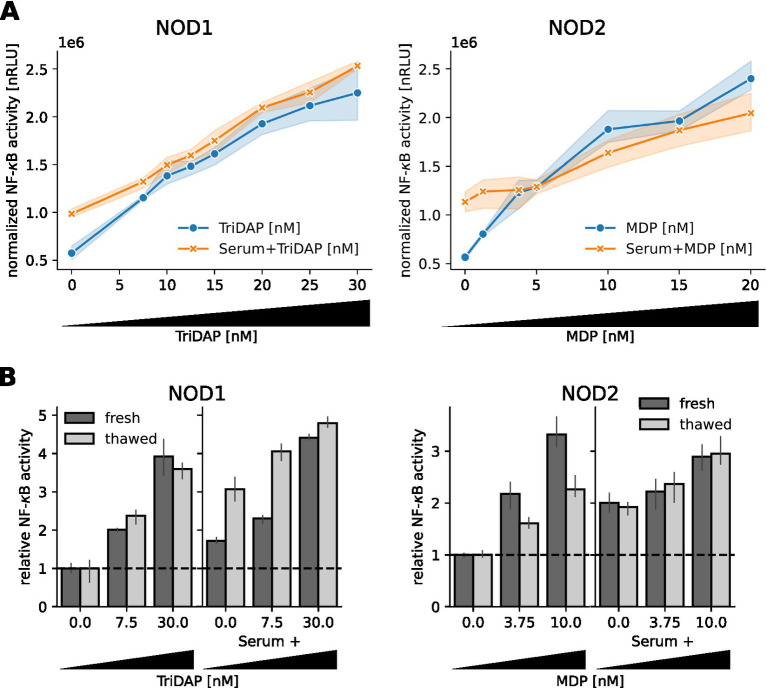
Sensitivity of the NOD1/2 assays. **(A)** HEK293T cells transfected with the NOD1 and NOD2 luciferase reporter system were stimulated 10 h after transfection with freshly drawn human serum spiked with different concentrations of TriDAP and MDP, additionally to different concentrations of TriDAP and MDP diluted in medium. NF-κB activity was normalized to the β-galactosidase signal and is shown as dots, together with the 95% confidence interval (*N* = 3). **(B)** Some samples were frozen at −80°C for three days to repeat the assay. NF-κB activity was normalized to unstimulated cells. Mean and SEM of one experiment measured in triplicates are shown.

To identify the lower detection limit of our modified luciferase-reporter assay, we stimulated NOD1 and NOD2 transfected cells with serum samples spiked with different concentrations of TriDAP and MDP. With both NOD1 and NOD2, we observed a dose dependent NF-κB response towards their respective ligand and the spiked serum samples. For the NOD1 luciferase-reporter assay, we observed a robust NF-κB activity increase with 7.5 nM TriDAP and serum spiked with 7.5 nM TriDAP (Figure 3A, left panel). The NOD2 assay detected already 1.25 nM MDP in medium and 10 nM MDP in serum (Figure 3A, right panel).

Samples from human subjects and from intervention studies are limited material and are typically stored frozen. To find out if freezing and thawing affected the bioactivity of TriDAP and MDP in serum, we froze spiked serum samples and diluted TriDAP and MDP in DMEM at −80°C for 3 days and repeated the assay. Surprisingly, we observed a slight increase in activity of the serum spiked with TriDAP (Figure 3B left panel), but no difference for the MDP spiked serum. MDP seems to be even stabilized by serum during freeze–thaw, as the NOD2 reactivity of the pure MDP dilutions was lower in the stored samples (Figure 3B, right panel).

Taken together, we could establish sensitive assays to measure NOD1 and NOD2 activity down to the low nM range of bacterial peptidoglycan. Our results show that freezing does not negatively affect NOD1/2 ligand activity in serum samples.

### Analysis of NOD1 and NOD2 activity in serum samples from a postprandial cohort

**Figure 4:**
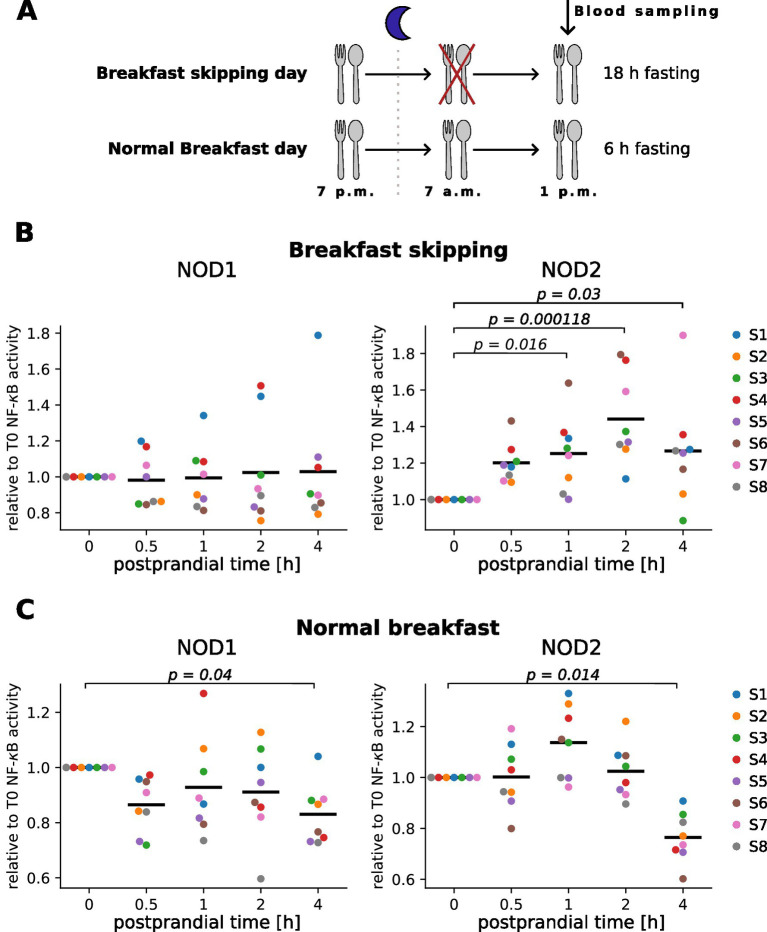
Measurement of NOD1 and NOD2 serum activity in human serum after food intake. **(A)** Schematic overview of the meal skipping study from Nas et al. (11). **(B,C)** HEK293T cells transfected with the NOD1 or NOD2 luciferase reporter system were stimulated 10 h after transfection with 10 μL of human serum from individuals that skipped breakfast **(B)** or received breakfast **(C)** before lunch. NF-κB activity was normalized to unstimulated cells and the preprandial value (T0). Each color represents one donor, and the mean of all individuals is shown as a line. Kruskal–Wallis test was performed followed by Dunn’s test if significant (*p*-value < 0.05). *p*-values from Dunn’s test are shown in the graphs. Experiments were performed in technical duplicates.

In order to evaluate our new assay protocol for detection of bacterial peptidoglycan in human serum samples, we used a series of serum samples from eight participants of the meal skipping study from Nas et al. (11). In this controlled intervention study, serum was taken before (0 h) and 0.5 h, 1 h, 2 h and 4 h after food intake. The study used lunchtime for the food challenge and a two-arm design including either a short (normal breakfast) or longer (breakfast skipping) fasting period directly before lunch (Figure 4A). Our results showed that NOD2 ligands increased in the serum of the participants already 0.5 h or 1 h after the food intake, compared to the preprandial condition (0 h) and peaked at 1 h or 2 h for skipped (Figure 4B, right panel) or normal breakfast sera (Figure 4C, right panel), respectively. A decline in the NOD2 reactivity was observed at the 4 h timepoint. Interestingly, the NOD1 and NOD2 signal of the normal breakfast serum decreased even below the preprandial level, whereas the breakfast skipped samples only showed reduction to about the levels measured at the 1 h timepoint (Figures 4B,C right panel). For NOD1 assay, interindividual differences were observed, however overall no clear trend or significant changes upon food intake were obtained (Figures 4B,C left panel).

To summarize, this shows that NOD2 ligands significantly change in the serum upon food intake and that fasting affects this kinetic.

## Discussion

Here we show that SEAP-based cell assays are not suitable for analysis of human serum, as the AP activity in the serum significantly affects the read-out (Figure 1). AP levels differ between individuals depending on age, sex, diseases or medication (14). In humans, AP levels are discussed as a marker for low-grade inflammation (15) and multiple diseases like the metabolic syndrome (16, 17). Additionally, individual AP serum levels can vary over the day, as they are decreased in a fasted state and increase 2 to 3 h after fat intake (18, 19), which further complicates the use of SEAP-based reporters in the context of postprandial reactions or metabolic diseases. This also applies to other mammalian species, namely mice (20). In some recent publications, such assays were used to show an increase in various systemic PRR ligands in high-fat diet fed mice (13) and as markers for small intestine barrier function in mice (21, 22) and men (22). To avoid that bias, only one study used heat treated (75°C for 5 min) serum, which inactivates AP activity. Using this serum, the authors observed an increase in TLR4, but not NOD2 ligands in mice with obesity (23). In other studies, this issue was not considered. In future works such shortcomings should be better addressed.

We propose that the assay protocol established here provides a suited and robust way to assay activity of NOD1 and NOD2 ligands in serum samples. This is corroborated by the validation of this protocol by spiked serum samples and in a serum sample cohort.

Moreover, in postprandial serum samples of the meal skipping cohort (11), we observed a postprandial increase of NOD2 ligands after lunch (Figure 4). As both PRRs are activated by peptidoglycan fragments that enter the circulation passively, we suspect that our NOD1 assay with a detection limit around 7.5 nM TriDAP might not be sensitive enough to detect the slight postprandial increase, beside potential biological differences between NOD1 and NOD2 ligand concentration in the serum. Another explanation could be that not only bacterial fragments but other molecules like dietary-specific fatty acids might be able to activate NOD2 after food intake (24, 25).

Interestingly, we observed a greater and faster increase of NOD2 ligands in the serum of the 18 h fasted participants (breakfast skipping day) compared to the 6 h fasted individuals (normal breakfast day) (Figure 4). It might be that fasting increases gut permeability, which was reported previously in mice (26) and rats (27). In the meal skipping study (11), the 18 h fasted participants showed higher variances in the postprandial response. Blood from breakfast skipping individuals showed phytohemagglutinin (PHA) induced IL-6 release that was significantly decreased below preprandial levels at 30 min and increased above preprandial levels 4 h postprandial (11). This may indicate an increased postprandial inflammation, which could be related to the elevated NOD2 ligands. However, additional cohorts, also using different meal intervention, should be tested to establish if this is a general mechanism.

On the other hand, NOD2 signaling was shown to be important for the functionality of the metabolism and to be protective against metabolic diseases like diabetes (7). In addition, NOD2 activating peptidoglycans in the circulation lowered blood glucose and reduced low grade inflammation in HFD fed mice. At the same time, an injection of NOD1 activating ligands reduced glucose tolerance (28). To this end, the postprandial increase of NOD2 ligands could also help the organism deal with the sudden increase of nutrients in the blood. Our results provide the basis to use this protocol for the analysis of serum samples and underlines a critical role of NOD2 ligands and NOD2 in the postprandial response.

## Data Availability

The original contributions presented in the study are included in the article/supplementary material, further inquiries can be directed to the corresponding author.
